# The ubiquitin ligase Triad1 influences myeloid leukemogenesis by regulating the integrated stress response

**DOI:** 10.1016/j.jbc.2025.110484

**Published:** 2025-07-16

**Authors:** Hao Wang, Francis Suh, Mariafausta Fischietti, Brian Wray, Szymon K. Filip, Peter A. Faull, Liping Hu, Weiqi Huang, Bin Liu, Leonidas C. Platanias, Elizabeth A. Eklund

**Affiliations:** 1 The Feinberg School of Medicine, Northwestern University, Chicago, Illinois, USA; 2 Jesse Brown VA Medical Center, Chicago, Illinois, USA; 3 Lurie Cancer Center, Northwestern University, Chicago, Illinois, USA; 4 Northwestern University Proteomics Core, Proteomics Center of Excellence, Northwestern University, Chicago, Illinois, USA

**Keywords:** E3 ubiquitin ligase, innate immunity, leukemia, ubiquitination, gene expression

## Abstract

Increased expression of a set of homeodomain transcription factors, including HoxA10, characterizes an adverse prognosis subtype of acute myeloid leukemia (AML). Examples of this subtype include AML with *KMT2A* or *MYST3*/*CREBBP* gene rearrangements, and an AML subset with normal cytogenetics. Previously, we identified *ARIH2*, the gene encoding Triad1, as a HoxA10 target gene. We determined that transcriptional activation of *ARIH2* by HoxA10 was necessary to terminate emergency granulopoiesis during the innate immune response but also antagonized leukemogenesis in a murine model of *KMT2A*-rearranged AML. Triad1 expression progressively decreases during the latent period preceding AML in this model, and Triad1 knockdown accelerates AML development. Triad1 is an E3 ubiquitin ligase, and we found that knocking down Triad1 decreased protein ubiquitination in myeloid cells. Therefore, proteins with Triad1-dependent ubiquitination might regulate leukemogenesis and/or the innate immune response. By proteomic screen, we identified Triad1-dependent ubiquitination of a set of proteins that regulate the integrated stress response (ISR), including Gcn1. The ISR prevents metabolic exhaustion during sustained inflammation by decreasing total mRNA translation and global protein synthesis, while altering the translatome to correct metabolic deficiencies and inhibit apoptosis. In cells with Triad1-knockdown, we defined a translatome consistent with ISR-activation and reversed by co-knockdown of Gcn1. Gcn1-knockdown also delayed AML development in a *KMT2A*-rearranged murine model, and reversed the effects of Triad1-knockdown on leukemogenesis. These results suggest ISR-inhibition mediates Triad1-related leukemia suppression, and activation of the ISR enhances leukemogenesis in this adverse-prognosis AML subtype.

Increased expression of a set of homeodomain (HD) proteins, including HoxA9 and HoxA10, is characteristic of AML with translocations or internal duplication of the *KMT2A* gene (also known as the Mixed Lineage Leukemia 1 gene; *MLL1*), translocation of the *MYST3*-*CREBBP* genes, or a normal cytogenetics subset with adverse outcomes (1, 2, 3, 4). We previously identified a set of target genes that are activated by cooperation between HoxA9 and HoxA10 and facilitate hematopoietic stem and progenitor cell (HSPC) expansion (5, 6, 7, 8). We also identified a set of target genes that are regulated by antagonism between HoxA9 and HoxA10, including *ARIH2*; the gene encoding Triad1. HoxA9 represses *ARIH2* transcription in HSPCs, but HoxA10 activates *ARIH2* in differentiating phagocytes (9, 10, 11, 12). A conserved HD tyrosine in HoxA9 and HoxA10 mediates this switch, with phosphorylation decreasing binding affinity of HoxA9 but increasing HoxA10 binding (9, 12). HoxA9 and HoxA10 are Shp2 substrates, and we found constitutive activation of this protein tyrosine phosphatase blocked *ARIH2* activation by HoxA10 and dysregulated the functional balance with HoxA9 (11, 12, 13).

Triad1 is an E3 ubiquitin (Ub) ligase that was initially identified in a screen for genes expressed during granulopoiesis (14, 15). We confirmed this expression profile and determined that Triad1-knockdown decreased overall protein ubiquitination in murine bone marrow progenitors or myeloid cell lines (9, 10). Triad1 overexpression in CD34^+^ cells decreases colony formation, and we found it was also required to terminate emergency granulopoiesis during the innate immune response (10, 11, 12). Steady state hematopoiesis is unremarkable in *HoxA9*^−/−^ or *HoxA10*^−/−^ mice, but *HoxA9*^−/−^ mice have impaired G-CSF-induced granulocytosis, while *HoxA10*^−/−^ mice exhibit an exaggerated and sustained emergency granulopoiesis response (10, 16, 17). The latter is corrected by re-expressing Triad1 in *HoxA10*^−/−^ marrow, implying that Triad1 regulates duration of the innate immune response (10).

AML develops with a latency of weeks in mice transplanted with bone marrow transduced with vectors to express MLL1-oncoproteins or to overexpress HoxA10, suggesting leukemogenesis requires additional mutations (4, 11, 12, 13). In murine recipients of bone marrow expressing the MLL1-ELL oncogene, we found Shp2-protein-tyrosine-phosphatase (PTP) activity increased during this latent period, with progressive decreases in phosphorylation of HoxA9 and HoxA10, and in Triad1 expression (11, 12). We also found co-transduction with vectors to express MLL1-ELL plus knockdown Triad1 shortened AML latency, suggesting Triad1 is a leukemia suppressor (11, 12). These murine studies suggest a multi-hit model for *KMT2A*-rearranged AML, consistent with findings in human leukemia. For example, in studies of twins with a common *KMT2A* mutation but discordant for AML, the twins with AML had acquired additional mutations, including *ARIH2* deletion (18).

In the current work, we screened for proteins with Triad1-dependent ubiquitination, since known Triad1 substrates do not explain all of the observed effects of Triad1 on leukemogenesis and the innate immune response. We identified proteins involved in the regulation of the innate immune response, as anticipated, but also in the activation of the ISR, a previously unknown Triad1 function. During the ISR, ribosome biogenesis and total mRNA translation decrease, resulting in a decrease in global protein synthesis. This provides some protection from the metabolic stress of sustained inflammation (19, 20, 21). To provide additional protection, the profile of translated messages (the translatome) is altered during the ISR to correct metabolic defects and prevent cell death. To accomplish this, the ISR translatome includes increased translation of proteins mediating amino acid or glucose import, lactate export, or inhibiting apoptosis (22, 23, 24). Enhanced Tp53 activity during the ISR facilitates cell cycle arrest for repair of stress-induced DNA damage, providing an additional layer of apoptosis protection (25).

In the current work, we identified Triad1-dependent ubiquitination and degradation of proteins that regulate the ISR, including Gcn1, eIF2b4, and eIF4g1 (26, 27). Amino acid starvation or oxidative stress activate Gcn1 with consequent activation of Gcn2; a key ISR mediator (26, 27). Gcn2 phosphorylates eIF2α, leading to inhibition of eIF2b and thus of global translation (28, 29, 30). The Gcn2/eIF2α complex induces Atf4 expression, expression of Atf4 target genes CHOP/DDIT3 and REDD1/DDIT4, and selective mRNA translation characteristic of the ISR (31, 32). We hypothesize E3 ubiquitin ligase activity of Triad1 inhibits the ISR during termination of the innate immune response. If ISR-inhibition is a physiologic counterpart of leukemia suppression, sustained ISR activation, due to decreased Triad1 during leukemogenesis, may contribute to disease progression in this molecular subset of adverse prognosis AML.

## Results

### Triad1 induces ubiquitination of proteins that regulate the ISR

Previously, we found that Triad1-knockdown decreased total protein ubiquitination in murine bone marrow progenitor cells or myeloid cell lines (9, 11). To screen for proteins with Triad1-dependent ubiquitination, we generated stable transfectant pools of U937 myeloid cells with Triad1-specific shRNA vectors or scrambled control shRNAs (11). Triad1-knockdown was consistently >70% by Western blot and quantitative real-time PCR (see below), and three independent pools for each set of constructs were separately analyzed for protein ubiquitination. To stabilize ubiquitinated proteins, transfectants were treated with proteosome and lysosome inhibitors before lysis (9). Lysates underwent enzymatic protein digestion, peptide desalting, and affinity chromatography with a ubiquitin branch (K-ε-GG) antibody column. Ubiquitinated peptides were identified and quantified by nano liquid chromatography mass spectrometry.

**Figure 1:**
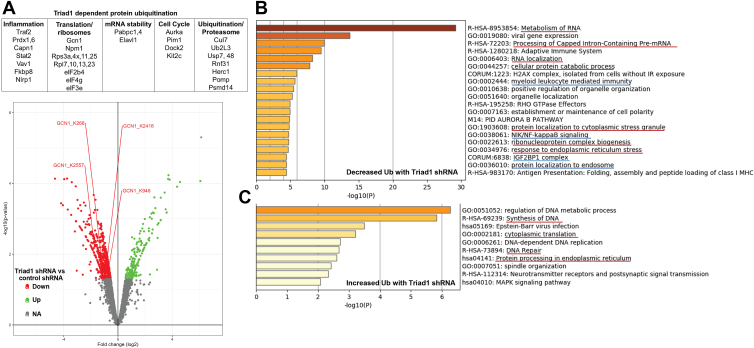
**Triad1 influences ubiquitination of proteins that regulate the ISR and the innate immune response.** U937 myeloid cells were stably transfected with vectors to express Triad1-specific shRNAs or scrambled shRNA controls, and lysates were screened for ubiquitinated proteins by affinity purification and mass-spectrometry. *A*, Triad1 influences ubiquitination of proteins that activate the ISR, including Gcn1. Statistically significant decrease in ubiquitination of Gcn1 residues with Triad1 knockdown *versus* controls is indicated (adjusted *p*-value < 0.05). Ubiquitinated protein sets were analyzed by gene ontology for functional pathways. By gene ontology analysis, Triad1 knockdown (*B*) decreases or (*C*) increases ubiquitination of proteins involved in pathways that contribute to the ISR (underlined in *red*) or the inflammatory response (underlined in *blue*).

We compared ubiquitinated peptides in cells with *versus* without Triad1-knockdown, and identified a set of novel proteins with statistically significant differences in ubiquitination (Fig. 1*A*). By gene ontology analysis, we defined molecular pathways associated with these proteins and thus potentially influenced by Triad1 ubiquitin ligase activity (33). We found that cells with Triad1-knockdown had a significant decrease (Fig. 1*B*) or increase (Fig. 1*C*) in ubiquitination of proteins involved in multiple cellular processes. This included some processes previously known to be influenced by Triad1 (underlined in blue), but some that were not. Many of the latter were in pathways relevant to the ISR (underlined in red). This included mRNA processing, ribosomal component biogenesis and assembly, translation, response to endoplasmic reticulum stress, and ISR initiation (20, 21, 22, 23, 24, 25, 34, 35).

Inhibition of ribosome component biogenesis and ribosome assembly is known to occur during the ISR (20). These processes and mRNA processing are facilitated by ubiquitination of component proteins, suggesting decreased ubiquitination impairs translation (21, 36). Conversely, ubiquitination and degradation of proteins involved in ER stress and the unfolded protein response may prevent apoptosis during the ISR (24). Gcn1, eIF2b4 and eIF4g1 also undergo ubiquitin-mediated degradation, and decreased ubiquitination of these proteins upon Triad1-knockdown is also consistent with a role in ISR regulation (Fig. 1*A*) (19). Ubiquitination of a small number of proteins increased in cells with Triad1-knockdown, consistent with our identification of decreased ubiquitination of other ubiquitin ligases or increased ubiquitination of deubiquitinases in these cells (Fig. 1*A*).

**Figure 2:**
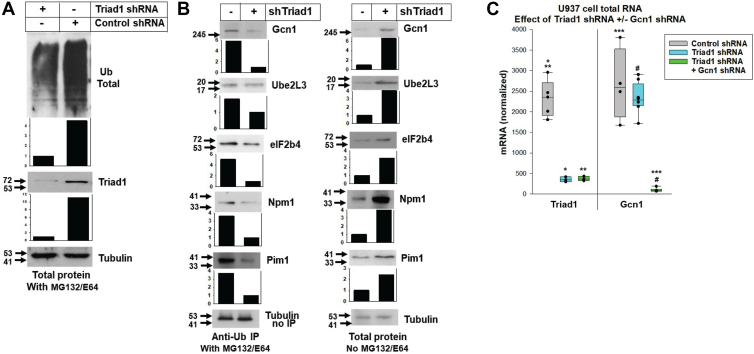
**Triad1-dependent ubiquitination destabilizes proteins identified by screening.** Lysates of independent stable transfectants were treated with lysosome or proteosome inhibitors and analyzed by anti-ubiquitin immunoprecipitation and Western blots. *A*, Triad1-knockdown decreases total protein ubiquitination in U937 myeloid cells. Cells were treated with MG132 or E64 to stabilize ubiquitinated proteins and Western blots of cell lysates were performed. Tubulin was a loading control and protein bands were quantified by densitometry (normalized to Tubulin). *B*, Triad1 increases ubiquitination and destabilizes proteins identified by screening. Panels on the *left* represent Western blots of anti-ubiquitin immunoprecipitants of MG132/E64 treated cell lysates, and on the *right* panels represent Western blots of total cell lysates without inhibitor treatment. Tubulin was a loading control and protein bands were quantified by densitometry (normalized to Tubulin). *C*, Gcn1 mRNA abundance is independent of Triad1-knockdown. RNA was quantified by real time PCR with actin as an internal control. Statistically significant differences indicated by ∗*p* = 0.003, ∗∗*p* = 0.005, ∗∗∗*p* = 0.007 or #*p* = 0.002 (by one way ANOVA with Tukey correction; n = 3 independent experiments performed in duplicate). Error bars represent SD and *p* < 0.05 considered significant.

We verified screening results in independent stable transfectant pools of U937 cells with Triad1-specific shRNAs or scrambled controls (duplicate samples from three independent pools were analyzed for each construct set). In initial experiments, we verified a decrease in total protein ubiquitination with Triad1-knockdown *versus* control, consistent with our prior results (Fig. 2*A*) (9, 11). We next assessed ubiquitination of selected proteins with statistically significant difference in ubiquitination with *versus* without Triad1-knockdown in our screen (Fig. 2*B*). Cells were treated with proteosome and lysosome inhibitors, and lysates analyzed by anti-ubiquitin immunoprecipitation followed by protein specific Western blot. Results were compared to total cell lysates from non-inhibitor treated cells (representative blots shown). We verified decreased ubiquitination and enhanced stability of Gcn1 protein in cells with Triad1-knockdown (Fig. 2*B*), without modulation of Gcn1 mRNA expression (Fig. 2*C*). Triad1 protein and mRNA were quantified in all transfectant pools by Western blot and real time PCR (Fig. 2, *A* and *C*).

### Effects of Triad1-knockdown on ribosome/polysome profiles are reversed by Gcn1-knockdown

Activation of the ISR by Triad1-knockdown is anticipated to decrease overall mRNA translation and global protein synthesis (19, 20). Therefore, we analyzed the effect of Triad1-knockdown on ribosome/polysome profiles in U937 stable transfectant pools, described above. To determine if the effects of Triad1-knockdown were due to Gcn1-stabilization, we generated stable transfectant pools with shRNA knockdown of both Gcn1 and Triad1. Gcn1-knockdown was >80% by Western blot (see below) and real-time PCR (*p* = 0.011 and *p* = 0.010, respectively, for comparison of cells with Gcn1-knockdown to those with scrambled shRNA control or Triad1-knockdown alone, by one-way ANOVA with Tukey correction, n = 6) (Fig. 2*C*). We similarly verified Triad1-knockdown in these transfectants (*p* = 0.0006 and *p* = 0.001, respectively, for scrambled shRNA control cells *versus* those with Triad1-knockdown or Triad1-plus Gcn1-knockdown, n = 6). We attempted to generate pools or clones with Gcn1-knockdown alone but were unable to select viable transfectants.

**Figure 3:**
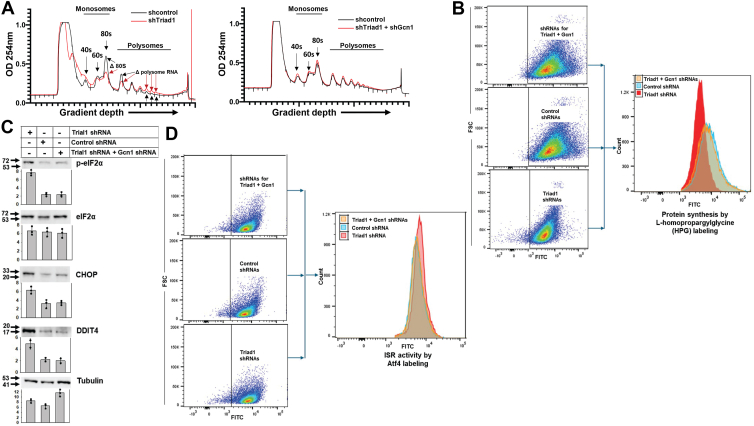
**The alterations of ribosome/polysome profiles, protein synthesis, and ISR activation due to Triad1-knockdown are reversed by Gcn1-knockdown.** U937 cells were stably transfected with vectors to express shRNAs to Triad1, Triad1 plus Gcn1, or scrambled controls. *A*, ribosome/polysome profiles of cells with Triad1-knockdown are normalized by Gcn1-knockdown. Monosomes, total polysomes and higher order polysomes are indicated. ISR activation by Triad1-knockdown was reversed by Gcn1 co-knockdown as indicated by reversal of (*B*) decreased global protein synthesis. Nascent proteins were quantified by flow cytometry of L-homopropargylglycine (HPG) labeled cells. A representative experiment is shown. *C*, phosphorylation of eIF4α and expression of CHOP/DDIT3 or REDD1/DDIT4. Western blots were performed with Tubulin as a loading control. Protein bands from three independent experiments were quantified by densitometry and normalized to Tubulin (indicated by •). Representative blots are shown. *D*, increased Atf4 abundance. Atf4 was quantified by flow cytometry, and a representative experiment is shown.

To generate ribosome/polysome profiles, transfectant lysates were analyzed on linear sucrose gradients to separate 40S and 60S ribosomal components from 80S ribosomes and higher-order polysomes. mRNAs are translated on 80S monosomes, but higher-order polysomes contain the most actively translated messages. We found that Triad1-knockdown decreased assembly of 80S ribosomes (Fig. 3*A*), consistent with the impact of ISR-activation on ribosome biogenesis/assembly and thus total mRNA translation (see Fig. 1*A*). The profile of higher-order polysomes was slightly altered, suggesting Triad1-knockdown modulates translation of a subset of messages. Compared to Triad1-knockdown alone, knockdown of Triad1 plus Gcn1 normalized the ribosome/polysome profile, consistent with mediation of some Triad1-knockdown effects by Gcn1 (Fig. 3*A*). Three independent pools were studied separately for each construct combination (representative tracing shown).

To study the impact of Triad1-knockdown on global protein synthesis, we studied these U937 stable transfectant pools for nascent protein production using L-homopropargylglycine labelling (HPG). With Triad1-knockdown, we found fewer nascent proteins were produced compared to transfectants with a scrambled control shRNA vector (by flow cytometry, note log scale, Fig. 3*B*). We found a wide distribution of staining intensity in control cells, with a higher mean value than cells with Triad1-knockdown (5.16-fold difference ± 0.44, *p* = 0.01, n = 3 for comparison between groups by one-way ANOVA with Tukey correction). Consistent with a role for Gcn1 in mediating this effect of Triad1-knockdown, HPG labeling was not significantly different in transfectants with knockdown of Triad1 plus Gcn1 compared to transfectants with control shRNA vectors (0.8-fold difference ± 0.17, *p* = 0.65, n = 3 for comparison between groups).

We used additional approaches to specifically assess the role of Triad1 in ISR activation in these U937 stable transfectant pools. In these studies, we determine eIF2α phosphorylation, expression downstream target Atf4, and of Atf4 target genes CHOP/DDIT3 and REDD1/DDIT4 (28, 31, 32, 37). In U937 cells with Triad1-knockdown, we found a 293% ± 33% increase in eIF2α phosphorylation (∼6 fold increase), a 98.6% ± 1.4% increase in CHOP expression, and a 106% ± 3.6% increase in REDD1 expression compared to control shRNA transfectants (by Western blot; *p* ≤ 0.001 for all three comparisons by one way ANOVA with Tukey correction, n = 3) (Fig. 3*C*). We also found that Triad1-knockdown induced a 178% ± 6.5% increase in Atf4 accumulation compared to shRNA control cells (by flow cytometry; *p* = 0.004, n = 3) (Fig. 3*D*).

To demonstrate the role of Gcn1 in ISR activation in cells with Triad1 knockdown, we compared U937 transfectants with knockdown of Triad1 plus Gcn1 to control shRNA transfectants. We found eIF2α phosphorylation, CHOP/DDTI3 and REDD1/DDTI4 expression, and Atf4 accumulation were not significantly different in cells with co-knockdown of Gcn1 plus Triad1 compared to control shRNA transfectants (*p* = 0.94 for phospho-eIF2α; *p* = 0.79 for CHOP/DDIT3; *p* = 0.88 for REDD1/DDTI4; and *p* = 0.72 for Atf4 by one-way ANOVA with Tukey correction, n = 3 for all comparisons).

### Effects of Triad1-knockdown on the translatome are reversed by Gcn1-knockdown

To identify Gcn1-dependent influences of Triad1 on the translatome, we sequenced polysome-associated mRNA from these stable transfectant pools. For each construct combination, we studied pooled polysome RNA from each of three independent stable transfectant pools (*i.e.*, three independent polysome RNA pools per construct combination). To identify polysome-specific differences in mRNA abundance, results were compared to total mRNA sequencing from the same cells. We compared transfectants with Triad1-knockdown, Triad1-plus Gcn1-knockdown, or scrambled shRNA controls. Statistically significant translatome differences were determined, and gene ontology analysis was applied to implicate pathway activity.

**Figure 4:**
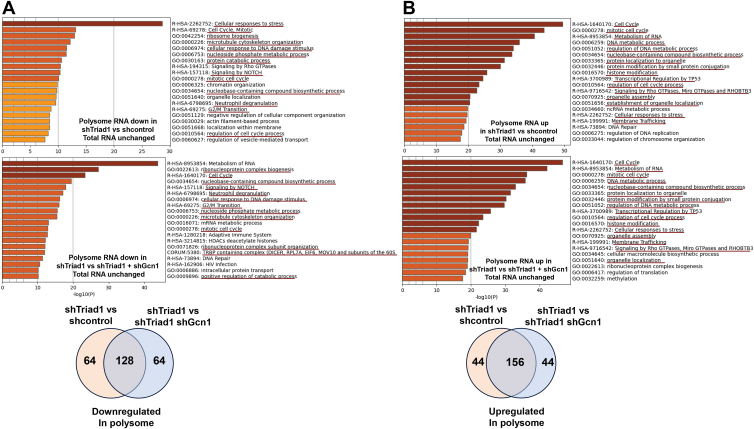
**Alterations in polysome-specific mRNA due to Triad1-knockdown were reversed by co-knockdown of Gcn1.** U937 cells were stably transfected with vectors to express shRNAs to Triad1, Triad1 plus Gcn1, or scrambled control. RNA sequencing of total mRNA *versus* polysome associated mRNA was performed to identify polysome-specific messages. Profiles were subjected to gene ontology analysis to identify pathways with (*A*) decreased or (*B*) increased translation with knockdown of Triad1 *versus* knockdown of Triad1 plus Gcn1 or scrambled controls. Pathways common to the two comparisons are underlined in *red*. Venn diagrams represent common genes with statistically significant differences in polysome-specific mRNA in transfectants with Triad1 shRNAs compared to those with shRNAs to both Triad1 and Gcn1, or with control shRNAs.

Consistent with inhibition of the ISR by Triad1, we found Triad1-knockdown decreased abundance of polysome-specific mRNAs involved in ribosome biogenesis, the DNA-damage response, and protein catabolism (Fig. 4*A*), but increased mRNAs regulating mitosis, DNA-repair, DNA and RNA metabolism, and Tp53 activity (Fig. 4*B*). Triad1-knockdown also increased polysome-specific mRNAs for membrane trafficking proteins, including solute carrier proteins involved in the import of amino acids, long-chain fatty acids, and glucose, but export of lactate and ions.

We compared polysome-specific mRNA profiles in cells with Triad1-knockdown to knockdown of Triad1 plus Gcn1, and identified a similar set of gene and pathway differences. Out of the top 200 genes with the greatest decrease in abundance of polysomal-specific mRNA in cells with Triad1-knockdown *versus* scrambled shRNA control, 64% were also in the first 200 polysome-specific genes with the greatest decrease with Triad1-knockdown *versus* Triad1-plus Gcn1-knockdown (Fig. 4*A*). Similarly, of the first 200 genes with the greatest increase in polysome-specific mRNA in cells with Triad1-knockdown *versus* scrambled shRNA control, 78% were the same as the top 200 polysome-specific genes in comparisons of Triad1-knockdown to Triad1-plus Gcn1-knockdown (Fig. 4*B*). There were minor differences in polysome-specific mRNA profiles in cells with Triad1-plus Gcn1-knockdown *versus* control cells, encompassing 20-fold fewer differences than comparisons of Triad1-knockdown *versus* Triad1-plus Gcn1-knockdown or shRNA control cells.

**Figure 5:**
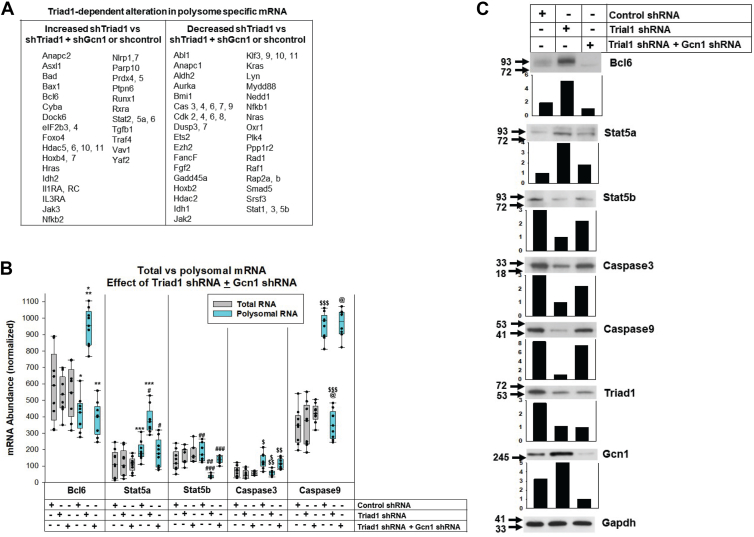
**Alterations in polysome-specific mRNA due to Triad1-knockdown, but reversed by Gcn1-knockdown, were reflected in protein expression.** U937 cells were stably transfected with vectors to express shRNAs to Triad1, Triad1 plus Gcn1, or a scrambled control. *A*, common sets of polysome-specific messages were altered in comparisons of Triad1 knockdown *versus* co-knockdown of Triad1 and Gcn1, or scrambled shRNA control. Statistical significance of differentially expressed genes was determined by an FDR-adjusted *p* < 0.05. *B*, polysome-specific messages of interest identified by RNA-sequencing were quantified by real-time PCR in independent transfectant pools. Statistically significant differences indicated by; ∗*p* = 0.004, ∗∗∗*p* = 0.001, #*p* = 0.002; ∗∗, ##, ###, $, $$, $$$ or @*p* < 0.001 (by one way ANOVA with Tukey correction; n = 3 independent experiments performed in triplicate). Error bars represent SD, and *p* < 0.05 is considered significant. *C*, Triad1/Gcn1-dependent translatome alterations were reflected in protein expression. Western blots of lysates were performed, and protein bands quantified by densitometry (normalized to Gapdh loading control).

We performed independent experiments with U937 stable transfectant pools to validate these screening results and investigate expression of proteins identified by polysome RNA-sequencing (Fig. 5*A*). We selected mRNAs with increased (Bcl6, Stat5a) or decreased (Stat5b, Caspase 3 or 9) polysome-specific abundance in the polysome mRNA screen with Triad1-knockdown *versus* either Triad1-plus Gcn1-knockdown or scrambled shRNA control. Three stable transfectant pools were generated for each combination, and polysome *versus* total mRNA was quantified (in triplicate).

We confirmed that knocking-down Triad1 or Triad1 plus Gcn1 did not alter total mRNA abundance for Bcl6, Stat5a, Stat5b, Caspase 3, or Caspase 9 compared to control cells (*p* = 0.81, *p* = 0.97, *p* = 0.38, *p* = 0.46, *p* = 0.20, respectively, by one way ANOVA with Tukey correction, n = 9). However, Triad1-knockdown increased abundance of polysome-associated Bcl6 and Stat5a mRNA compared to combined knockdown of Triad1 plus Gcn1 (*p* = 0.0004 and *p* = 0.0001, respectively, for Bcl6 and Stat5a, n = 9) (Fig. 5*B*). Polysome-associated Bcl6 and Stat5a mRNA abundance was not different in cells with knockdown of both Triad1 and Gcn1 compared to shRNA control cells (*p* = 0.52 and *p* = 0.78, respectively, for Bcl6 and Stat5a, n = 9). Conversely, there was less Stat5b, Caspase 3 or 9 polysomal mRNA in transfectants with Triad1-knockdown compared to combined knockdown of Triad1 plus Gcn1 or shRNA control cells (*p* < 0.001 for all comparisons, n = 9), but the latter two were not significantly different (*p* = 0.79, *p* = 0.27 or *p* = 0.77, respectively, for Stat5b, Caspase 3 or 9, n = 9) (Fig. 5*B*). We found these translatome differences paralleled differences in protein expression, suggesting functional significance to Triad1/Gcn1-dependent alterations in polysomal-specific abundance (Fig. 5*C*).

In control experiments, we found efficient Triad1-knockdown in these transfectants, with or without co-knockdown of Gcn1 (Fig. 5*C*). And, Triad1-knockdown alone increased Gcn1 protein compared to control cells (5.2-fold increase ± 0.34, *p* = 0.005, n = 4 different blots by one-way ANOVA with Tukey correction). Each experiment was performed using three independent transfectant pools for each construct combination, and representative Western blots are shown.

### Gcn1-knockdown reverses the effect of Triad1-knockdown on latency to MLL1-oncogene induced AML

**Figure 6:**
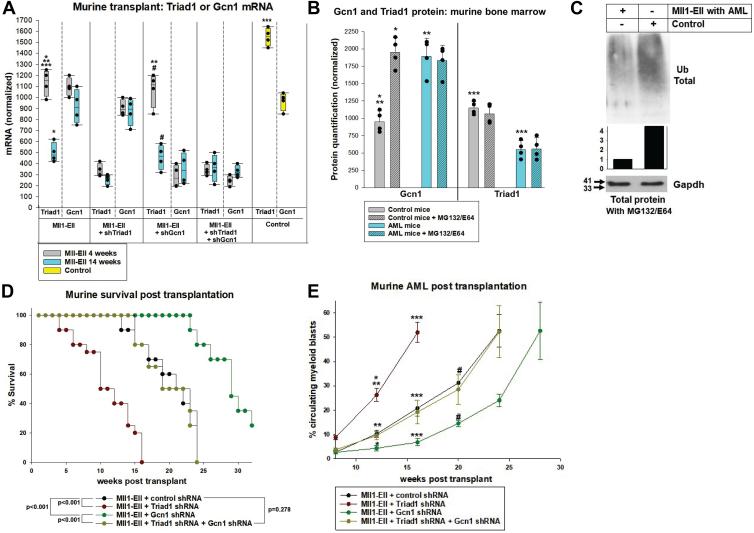
**Gcn1-knockdown reverses effects of Triad1-knockdown on latency to MLL1-ELL-induced AML.** Mice were transplanted with bone marrow transduced with MLL1-ELL expression vector plus shRNA vectors to knockdown; Triad1, Gcn1, Triad1 plus Gcn1, or scrambled control vectors. *A*, Triad1, but not Gcn1, mRNA decreases during MLL1-ELL-induced leukemogenesis. GFP^+^Lin^−^ bone marrow cells were isolated early *versus* late post-transplant, and Triad1 or Gcn1 quantified by real time PCR. Differences indicated by; ∗ or #*p* < 0.001, ∗∗*p* = 0.54, or ∗∗∗*p* = 0.001 (by one way ANOVA with Tukey correction; n = 4 mice per group). Error bars indicate SD and *p* < 0.05 considered significant. *B*, Gcn1-ubiquitination decreases during AML progression. Cells were treated with lysosome and proteosome inhibitors (MG132/E64) *versus* vehicle control and lysates analyzed by Western blot. Protein abundance was quantified and statistically significant differences indicated by ∗, ∗∗ or ∗∗∗*p* < 0.001 (by one way ANOVA with Tukey correction; n = 4 mice per group). Error bars indicate SD and *p* < 0.05 considered significant. *C*, total protein ubiquitination is decreased in the bone marrow of mice with established MLL-ELL induced AML. Lin^−^ckit^+^ bone marrow cells were treated with proteosome and lysosome inhibitors and lysates immunoprecipitated with anti-ubiquitin antibody and analyzed by Western blot. Protein bands were quantified by densitometry (normalized to Gapdh loading control). *D*, survival of mice transplanted with MLL1-ELL-transduced bone marrow is decreased by Triad1-knockdown but increased by Gcn1-knockdown. Statistically significant differences are indicated within the figure (by LogRank Analysis; n = 10 mice per group). *E*, latency to MLL1-ELL-induced AML is increased by Triad1-knockdown, but decreased by Gcn1-knockdown. AML is defined as >20% circulating myeloid blasts. Statistical significance is indicated by; ∗*p* = 0.044, ∗∗*p* < 0.001, ∗∗∗*p* = 0.020 or #*p* = 0.016 (by one way ANOVA with Tukey correction; n = 10 mice per group). Error bars indicate SD and *p* < 0.05 considered significant.

AML develops after a lag time of weeks in mice transplanted with bone marrow transduced with MLL1-oncogenes, but we found Triad1-knockdown significantly shortens this latency, suggesting it is a leukemia suppressor (3, 11, 12). To determine the contribution of Gcn1-degradation to leukemia suppression by Triad1, we co-transduced murine bone marrow cells with an MLL1-ELL expression vector plus vectors to express Triad1-specific shRNAs, Gcn1-specific shRNAs, Triad1-plus Gcn1-specific shRNAs, or scrambled controls. Bone marrow was transplanted into lethally irradiated syngeneic mice (11, 12). We quantified Triad1-and/or Gcn1-knockdown in GFP^+^Lin^−^ bone marrow cells from recipients collected at various timepoints (Fig. 6*A*). These studies confirmed a decrease in Triad1 during AML latency in recipients of MLL1-ELL-transduced bone marrow and verified that Gcn1 mRNA was not altered by Triad1-knockdown or the time post-transplant.

We next determined if decreased Triad1 during leukemogenesis stabilized Gcn1 by decreasing ubiquitin-mediated degradation *in vivo*. We compared GFP^+^Lin^−^ bone marrow cells from mice with established MLL1-ELL-AML to the same population from control mice. Some cells were pre-treated with proteasome and lysosome inhibitors to stabilize ubiquitinated proteins, and lysates were analyzed for Gcn1 or Triad1 by Western blot. In cells from recipients of control vector transduced marrow, we found proteasome/lysosome inhibitor treatment increased Gcn1 protein (*p* < 0.001, n = 4 by one-way ANOVA with Tukey correction) (Fig. 6*B*). In contrast, in MLL1-ELL-transduced bone marrow from recipients with established AML, Gcn1 protein was elevated without proteasome and lysosome inhibitors, and did not increase with this treatment (*p* = 0.07, n = 4) (Fig. 6*B*). Consistent with these results, total protein ubiquitination was less in recipients of MLL1-ELL transduced marrow with fully developed AML compared to control cells, as in our prior work (Fig. 6*C*). Experiments were performed with bone marrow from four mice per cohort, and proteins on Western blots were quantified by densitometry.

We also studied the impact of Triad1-and/or Gcn1-knockdown on the survival of recipients of MLL1-ELL-transduced bone marrow. As anticipated, survival was shorter in recipients of marrow transduced with MLL1-ELL plus Triad1-specific shRNA *versus* MLL1-ELL alone (50% survival 12 ± 3 weeks *versus* 23 ± 4 weeks, *p* < 0.001, n = 10 by LogRank Analysis) (Fig. 6*D*) (11). However, survival in recipients of marrow with MLL1-ELL plus both Triad-specific shRNA and Gcn1-specific shRNA vectors was not different than MLL1-ELL alone (50% survival in either group was ∼22 weeks; *p* = 0.278, n = 10). Additionally, survival was increased in recipients of marrow with vectors to express MLL1-ELL plus Gcn1-specific shRNAs compared to MLL1-ELL alone (50% survival, 29 ± 3 *versus* 21 ± 2 weeks, *p* < 0.001, n = 10).

These survival differences reflected AML latency, defined as time to ≥20% circulating myeloid blasts (Fig. 6*E*) (11, 12). Latency was not significantly different in recipients of bone marrow with MLL1-ELL plus combined knockdown of Triad1 and Gcn1 *versus* MLL1-ELL alone (*p* = 0.78 at 12 weeks, *p* = 0.74 at 16 weeks, *p* = 0.66 at 20 weeks, by one-way ANOVA with Tukey correction, n = 6). Circulating blasts increased earlier in recipients of marrow with MLL1-ELL plus Triad1 shRNA compared to other groups, as anticipated (*p* < 0.001 at 12 or 16 weeks, n = 6). Conversely, myeloid blasts increased later in recipients of marrow with MLL1-ELL plus Gcn1 shRNA *versus* MLL1-ELL alone (*p* = 0.049 at 12 weeks, *p* = 0.024 at 16 weeks, *p* = 0.012 at 20 weeks, n = 6).

## Discussion

Our prior studies indicated that Triad1 is required to terminate emergency granulopoiesis and has leukemia-suppressor activity in *KMT2A*-rearranged AML (10, 11, 12). To identify molecular mechanisms for these effects, we screened myeloid cells for Triad1-dependent protein ubiquitination. In the current work, we identified ISR-inhibition, *via* ubiquitination/degradation of Gcn1, as a novel Triad1 function. This is a previously unknown mechanism for the regulation of Gcn1, and since Gcn1 activates the key ISR-activator Gcn2, it implicates Triad1 in ISR regulation. We also identified Triad1-dependent ubiquitination of inflammatory modulators, including Traf2, calpains, and peroxiredoxins. These Triad1 effects may be at the interface between inflammation and leukemogenesis, and are of interest for additional study.

With our experimental design, ubiquitination of proteins identified by screening may be influenced directly or indirectly by Triad1. The latter may be substrates for ubiquitin ligases or de-ubiquitinases that are activated or degraded consequent to Triad1-induced ubiquitination (see Fig. 1*A*). Both categories of Triad1-influenced proteins are of interest. Since our goal was to identify pathways that are functionally relevant to leukemogenesis, we focused on defining the significance of a pathway including HoxA10, Triad1, and activation of the ISR by Gcn1.

We found that Triad1-knockdown in myeloid cells decreased total protein ubiquitination and modified ubiquitination of proteins involved in ISR-relevant events including; inhibition of ribosomal biogenesis (assembly requires component ubiquitination), modulation of DNA repair (DNA-damage response requires regulatory ubiquitination), inhibition of the unfolded protein response (resulting in apoptosis resistance), and mRNA processing and metabolism (enhanced or inhibited by ubiquitination of various proteins) (20, 21, 22, 23, 24, 25, 36, 38). Consistent with the hypothesis that Triad1 inhibits the ISR, we found increased eIF2α phosphorylation and abundance of Atf4, CHOP/DDIT3, and REDD1/DDIT4 proteins in cells with Triad1-knockdown, with a decrease in 80S ribosome assembly and *de novo* protein synthesis (28, 31, 32, 37). The translatome in these cells was also consistent with activation of the ISR, including enhanced translation of messages involved in apoptosis resistance, Tp53-mediated cell cycle arrest, and membrane trafficking to correct metabolic defects, but decreased translation of messages involved in ribosome biogenesis, protein catabolism, and the TRBP complex.

Consistent with mediation of Triad1 effects by Gcn1 degradation, combined Triad1-plus Gcn1-knockdown in U937 cells reverted events associated with ISR activation, the ribosome/polysome profile, and the translatome to resemble control cells. In cells with Triad1-knockdown, alteration in key polysome-specific mRNAs correlated with protein expression, functionally connecting HoxA10, Triad1, Gcn1, translation, and protein abundance. We were unable to study U937 cells with Gcn1-knockdown alone due to impaired cell survival during selection. However, we were able to knock down Gcn1 in MLL1-ELL transduced murine bone marrow for an *in vivo* study.

Endogenous Triad1 decreases during the lag time to AML in recipients of MLL1-ELL-transduced bone marrow, and Triad-knockdown accelerates leukemogenesis in this model (11, 12, 13). We previously found that Triad1-dependent ubiquitination/degradation of receptor tyrosine kinases, including Fgf-R1, contributed to leukemia suppression (12). If the ISR enhances AML progression, a decrease in Triad1 during AML latency may contribute to MLL1-ELL-induced leukemogenesis *via* Gcn1-stabilization. Consistent with this, Gcn1-knockdown reversed the effect of Triad1-knockdown on the *in vivo* progression of MLL1-ELL-AML. And, Gcn1-knockdown in MLL1-ELL-transduced bone marrow increased AML latency and survival, suggesting a role for Gcn1 and the ISR in leukemia progression. This is of interest, since small molecule inhibitors of Gcn1 and 2 were recently described (39, 40). The ISR has not been previously associated with protection from metabolic stress of leukemogenesis in this adverse prognosis AML subset.

These studies suggest the ISR translatome facilitates AML progression. In mice with MLL1-ELL-induced AML, we found decreases in Triad1, protein ubiquitination, and Gcn1 ubiquitination/degradation. Gcn1-stabilization increased translation of anti-apoptotic proteins Bcl6 and Nfkb2, signal modulators Stat5a and Shp1, and the emergency granulopoiesis mediator IL1R but decreased translation of Caspases, Fanconi DNA repair proteins, and leukemia suppressors Ezh2 and Idh1 ((13, 41, 42, 43)). The role of translational modulation of these proteins in AML is of interest.

## Experimental procedures

### Plasmids and cell lines

Plasmids with Gcn1-specific or scrambled shRNAs were obtained from OriGene (Rockville, MD), and with Triad1-specific shRNAs generated as described (9, 11). We used combinations of three different shRNA vectors each to knockdown Gcn1 or Triad1 for comparison to scrambled controls. The shRNAs in these vectors represented sequences in different cDNA regions. MLL1-ELL cDNA was obtained from D-E. Zhang (University of California) and subcloned into the MiGR1 vector (AddGene).

U937 cells were obtained from A. Kraft (University of Arizona), and 293T and NIH3T3 cells from ATCC. Lines were annually validated by genomic fingerprinting (ATCC Whatman FTA Human STR Kit). U937 cells were transfected by electroporation (Biorad) with shRNA vectors to knock down Triad1 and/or Gcn1 (*versus* scrambled controls). Stable transfectant pools were selected in G418 and/or puromycin and verified by Western blots and real-time PCR (9). At least three independent pools were used for all studies.

### Screening for ubiquitination

U937 stable transfectant pools were treated with proteosome and lysosome inhibitors for 24 h (MG132 = 2.5 μM and E64 = 5 μM) (9). Cells were lysed in urea buffer, and 10 mg of protein per sample was reduced with dithiothreitol, alkylated with iodoacetamide, then digested with Lys-C and trypsin (Promega) overnight at 37 °C. Peptides were desalted with C18 SepPak columns (Waters), and eluates were lyophilized to dryness. Ubiquitinated peptides were enriched using PTMScan Ubiquitin Remnant Motif (K-ε-GG) Kit (Cell Signaling Technology) per the manufacturer’s protocol. Eluates were dried by vacuum centrifugation, then resuspended in 25 μl of 0.1% aqueous formic acid, shaken and sonicated for 10 min each, and centrifuged for 5 min at 20,000*g*. 25 μl of peptide supernatant was transferred to a glass autosampler vial, then placed in the autosampler of a Thermo Scientific Ultimate 3000 nano liquid chromatography instrument. 4 μl of sample was loaded onto an in-house packed C18 trap column using a 2.5 μl/min flow rate (0.1% aqueous trifluoroacetic acid) then transferred to a C18 analytical column (75 μm × 25 cm). Peptides were separated using an increasing gradient of organic mobile phase (80% acetonitrile and 0.1% aqueous formic acid) and nanoelectrosprayed into the heated source of a Thermo Scientific QExactive HF mass spectrometer. The 20 most abundant peptides per survey (MS1; 60,000 resolution, 3E6 AGC, 120 ms maximum injection time) scan were isolated and fragmented (MS/MS; 30,000 resolution, 1E5 AGC, 120 ms maximum injection time, HCD fragmentation, 20 s dynamic exclusion) over a 180-minute method. Proteomic screening was performed in collaboration with the Northwestern University Proteomics Core. Gene ontology of ubiquitinated proteins was determined in collaboration with the Lurie Cancer Center Quantitative Data Sciences Core.

### Proteomics data processing and analysis

Twelve raw files were processed using FragPipe (v22.0) with the LFQ-Ubiquitin search workflow. During processing, technical replicates were merged, yielding three samples for scrambled control shRNA and four for Triad1 shRNA. Data was searched against the SwissProt human database (downloaded on September 30, 2024). Variable modifications included N-terminal acetylation (+42.0106 Da), GlyGly modification on lysine (+114.04293 Da), and methionine oxidation (+15.9949 Da). PTMProphet and MSBooster were enabled, with peptide scoring performed using the Koina server (https://koina.wilhelmlab.org:443/v2/models/). The output file, “combined_site_K_114.0429.tsv”, containing GlyGly-modified lysine residues, was analyzed using R(v4.4.2) with the limma package, accounting for mass spectrometry run batches as a confounding factor. Volcano plots were generated to visualize each modification site using both unadjusted and adjusted *p*-values.

### Polysome RNA isolation and profiling

U937 transfectants were treated with cycloheximide (100 μg/ml) and lysed in column buffer (44). To separate ribosomal fractions, lysates were layered on a 5 to 50% sucrose gradient and centrifuged 120 min at 35,000 rpm in a Beckman SW41-Ti rotor (Pasadana, CA). Absorbance was continuously measured at 254 nm with an ISCO density gradient fractionator (Brandel, Gaithersburg, MD) (44). Fluorinert TM FC-40 (Sigma-Aldrich) was used to set the detector baseline. RNA was isolated from pooled polysomal fractions for comparison to total input RNA (AllPrep RNA/Protein Kit, Qiagen).

### RNA sequencing

Stranded RNA-Seq of total *versus* polysomal RNA was conducted in Northwestern University’s Center for Genetic Medicine. RNA quality was determined using an Agilent Bioanalyzer 2100. Libraries were prepared using the Illumina TruSeq Stranded mRNA kit. Single-end, 75 bp reads were generated with an Illumina NextSeq 500 Sequencer. DNA read quality was evaluated by FastQC. Adapters were trimmed, and reads of poor quality or aligning to rRNA were filtered. Cleaned reads were aligned to the human genome using STAR, and read counts were calculated by htseq-count. Differential expression was determined with DESeq2 (45). Statistical significance of differentially expressed genes was determined by an FDR-adjusted *p*-value < 0.05.

### Protein and mRNA analysis

Western blots were performed with antibodies to Triad1, eIF2b4, Pim1, Stat5a, Caspase 3, Caspase 9, CHOP/DDIT3, Tubulin or Gapdh (Proteintech), Gcn1 or Ube2L3 (Abcam), REDD/DDIT4 (Invitrogen), or Bcl6, Stat5b, or total and phospho-eIF2α (Cell Signaling Technology). Some cells were treated with lysosome and proteosome inhibitors prior to lysis. Lysates were immunoprecipitated with anti-ubiquitin antibody (Cell Signaling Technology) followed by Western blot (9). Bands on Western blots were quantified using ImageJ 1.54 g (Wayne Rasband and contributors, National Institutes of Health, Bethesda, MD).

RNA was isolated with Triazol reagent and quantified by real-time PCR using the standard curve method (9). Primers were designed with the Promega website.

### Flow cytometry

Nascent protein synthesis was quantified using the “Click-It HPG” system to stain for L-homopropargylglycine (HPG) incorporation into proteins (per manufacturer’s instructions; Life Technologies). For this assay, U937 stable transfectants were incubated with HPG in methionine-free media, followed by incubation with Alexa Fluor 594 azide to ligate green-fluorescent azide to the incorporated alkyne. Cell labeling was quantified by flow cytometry.

To assay for ISR activation, stable transfectant pools were incubated in serum-free media followed by fixation and permeabilization using the “Intracellular Fixation and Permeabilization Buffer Set” (per manufacturer’s instructions; eBiosciences). Permeabilized cells were labeled with FITC-conjugated Atf4 polyclonal antibody (Proteintech), and cell labeling was quantified by flow cytometry.

### Murine bone marrow transduction

For retroviral production, 293T cells were transfected with plasmids by electroporation, supernatants collected 48 h later, and virus titrated in NIH3T3 cells (10, 11, 12, 13). For retroviral transduction, bone marrow mononuclear cells from C57/Black six mice (Jackson Labs, Farmington, CT) were cultured in DMEM, 10% fetal bovine serum, 1% penicillin-streptomycin, 20 ng/ml GM-CSF, 20 ng/ml IL-3 and 100 ng/ml SCF (R&D Systems, Minneapolis, MN) and incubated with retroviral supernatant (∼10^7^ PFU/ml) plus polybrene (6 μg/ml) (10, 11, 12, 13). Mll1-Ell transduction efficiency was determined by flow cytometry for green fluorescent protein (GFP). Mll1-Ell expression and Triad1 or Gcn1 knockdown were confirmed by real-time PCR.

### Murine bone marrow transplantation

Transduced, viable Lin^−^ murine bone marrow cells were isolated by magnetic bead affinity (Miltenyi Biotech, Auburn, CA). Lethally irradiated syngeneic mice were injected with GFP^+^Lin^−^Sca1^+^ cells (2 × 10^5^) (11, 12). Ten mice were studied per group for an 80% chance of detecting a 20% difference between groups. No mice were excluded from evaluation. No randomization or investigator “blinding” was required in the study design. Peripheral blood counts were determined using a Hemavet counter (Erba Diagnostics).

### Statistical methods

Significance was determined one way ANOVA with Tukey correction using SigmaPlot (Grafiti LLC). Data is reported as average ± SD, with *p* < 0.05 considered significant. Survival/relapse rate differences were analyzed by Log-Rank analysis. Blood counts in treatment cohorts were analyzed for variance within groups *versus* between groups by Kruskal-Wallis One-Way Analysis of Variance on Ranks.

### Murine studies

Approved by the IACUC of Northwestern University and Jesse Brown VA.

## Data availability

Proteomics data were deposited to the ProteomeXchange Consortium (http://proteomecentral.proteomexchange.org) (46) with dataset identifier PXD060787. Total RNA-Seq data is available in the GEO repository (GEO Submission identifier GSE284048). All other data is provided in the manuscript with original data available from e-eklund@northwestern.edu.

## Conflict of interest

The authors declare that they have no conflicts of interest with the contents of this article.
